# A Content Review of Articles Published in the Medical Surveillance Monthly Report, 2019–2023

**Published:** 2024-01-20

**Authors:** Kristen R. Rossi, Robert N. Pursley

In continuous publication since 1995, the *Medical Surveillance Monthly Report (MSMR)* serves as the official peer-reviewed journal of the Armed Forces Health Surveillance Division (AFHSD) and the Defense Health Agency (DHA) Public Health Directorate. This monthly publication provides evidence-based estimates of the incidence, distribution, impact, and trends of illness and injury among U.S. military service members and associated populations. *MSMR* reports present data, public health information, and original research with direct relevance to the operational fitness of military members or Military Health System (MHS) beneficiaries’ health, safety, and well-being.

This editorial provides a bibliometric summary and thematic analysis for articles published in *MSMR* over a 5-year period, from January 2019 through December 2023. The bibliometric summary provides annual metadata on most-read articles and journal impact, while the thematic content analysis reviews published article data to quantify the populations of focus and primary study outcomes or topics of interest, which are further grouped into major thematic categories, corresponding to the International Classification of Diseases (ICD) chapter subjects. For analytic summaries described in full and brief reports as well as Surveillance Snapshots, the data sources utilized to study the main outcome were also reviewed.

## Summary Data

From January 2019 through December 2023, *MSMR* published a total of 248 articles. Full reports (n=166), Surveillance Snapshots (n=33), and brief reports (n=17) were the predominant content types, followed by a range of other articles including editorials (n=10), outbreak reports (n=7), case reports (n=6), and historical perspectives (n=5). Less-employed article types included letters to the editor (n=2), notice to readers (n=1), and Images in Health Surveillance (n=1).

**Figure (1):**
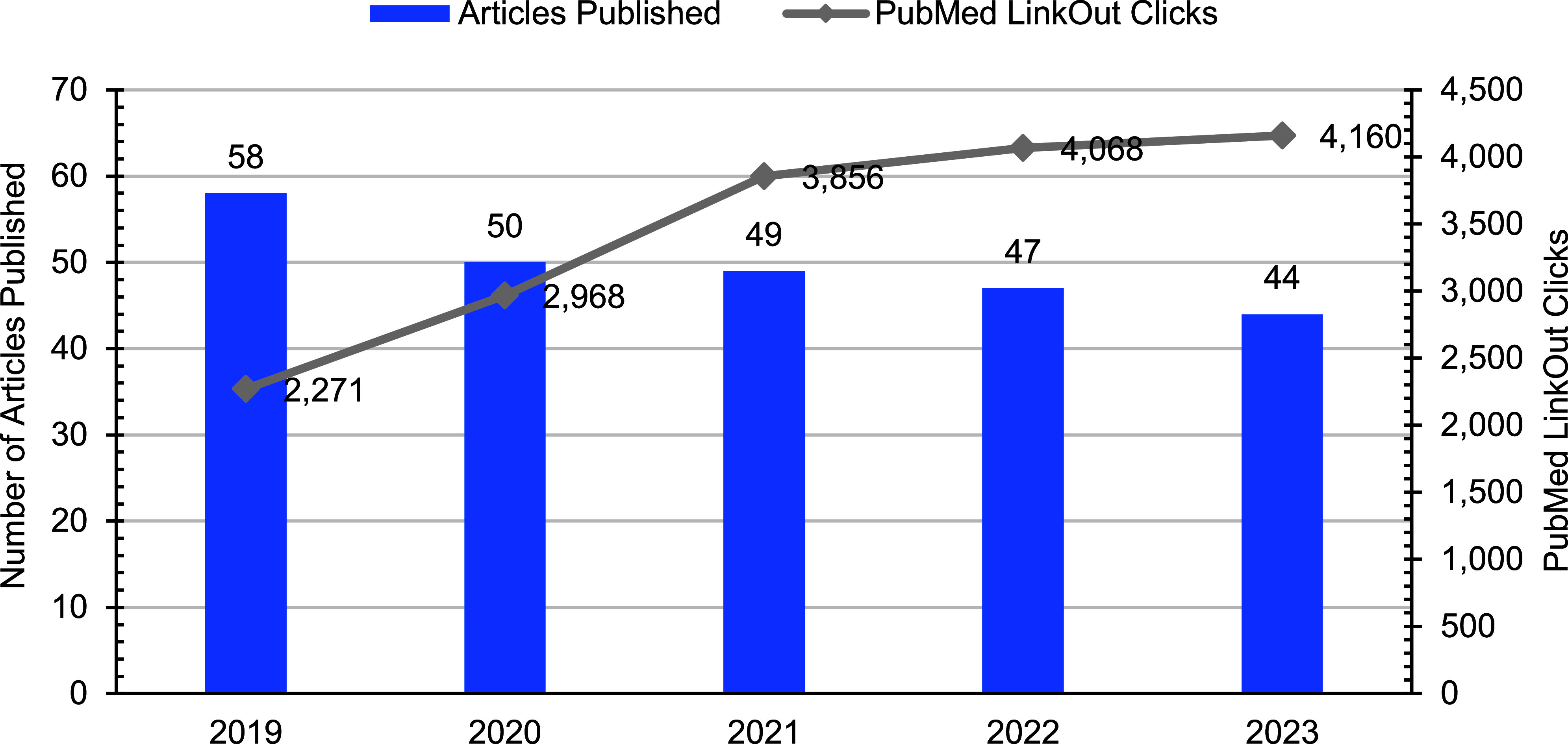
Annual Bibliometric Summary Data, 2019–2023.^a^ ^a^PubMed LinkOut Hits represent the annual number of LinkOut 'hits' (clicks) on the publisher’s icons in PubMed’s abstract display and clicks on the publisher’s links in the LinkOut list of resources.

The annual number of published articles declined over the past 5 years, from 58 articles in 2019 to 44 articles in 2023 (**Figure 1**). Notably, a summary of Reportable Medical Events (RMEs) for Department of Defense (DOD) service members and other MHS beneficiaries returned to *MSMR* in 2023; however, the 8 summary reports published in 2023 were not included for this content review. Sentinel RME summaries were regular features of *MSMR* until 2010. [1]

The population of interest in the majority of articles (n=206; 83.1%) focused on service members (active component, reserve component, or former service members), while 9.6% (n=24) of articles centered on both service members and non-service member beneficiaries, and 5.6% (n=14) were limited to non-service member beneficiaries; 4 articles did not include a particular population of interest (e.g., environmental sampling data).

## Content Themes

The content themes for each *MSMR* article published over the 5-year period were reviewed and then grouped into major thematic categories corresponding to ICD chapter subjects. Content themes not aligned to a chapter subject group were classified into separate categories, described in Table 1. Among the 248 total articles published in *MSMR* from 2019 to 2023, 42 (16.9%) articles provided a summary of total health care burden and provision, rather than 1 specific thematic topic. Each year, the *MSMR* publishes an annual compendium of burden of disease reports that groups diagnoses to inform readership of the major drivers of health care provision within the MHS. [2]

 While injuries, musculoskeletal diseases, and mental health disorders are the categories of medical conditions associated with most medical encounters and greatest numbers of hospital bed days reported among active component service members in 2022,2 infectious and parasitic diseases (n=73, 29.4%) represented a substantial majority of publication topics identified in the 5-year content theme review, described in **Table 1**. Additionally, maternal conditions contributed to approximately 13% of all hospital bed days for active component female service members in 2022, [2] but no articles were published for service women on the topics of pregnancy, childbirth, and the puerperium, or for certain conditions originating in the perinatal period.

Publication themes discussing contact with health services or procedures (‘Z-codes’) represented 24 (9.7%) of all articles; the majority of these were related to immunization (n=14, 5.6%) (**Table 1**). Other topics associated with health service contacts or procedures (n=10, 4.0%) included women’s health issues related to contraception use, infertility, menstrual suppression, or cervical cancer screening (n=5,) as well as men’s health issues related to vasectomy or testosterone replacement therapy (n=2) (data not shown).

## Data Sources

The methods of each article were manually reviewed to classify the data source of the major outcome of interest that was described in full and brief reports as well as Surveillance Snapshots (n=216). Six data source categories were assessed for each article, including: 1) administrative inpatient and ambulatory records, 2) laboratory results, 3) pharmacy prescriptions, 4) RME records, 5) survey data, and 6) all other data. Many articles included more than 1 data source for the major outcome of interest, and each of those data sources were classified independently. Since immunization records may be stored in a range of service-specific data systems, or as administrative records, those data were included in the ‘other data source’ category. Data outside of the main outcome of interest that were related to covariate or dependent variable analyses were not assessed.

Almost 30% (n=61) of full and brief reports and Surveillance Snapshots combined more than 1 data source for analysis of an outcome of interest. Administrative data for clinical conditions from inpatient and ambulatory records, based on ICD diagnoses, contributed to a substantive majority of articles (n=150, 69.4%), followed by laboratory data (n=44, 20.4%), RME records (n=42, 19.4%), then other data sources (n=45, 20.8%). Survey data (n=14, 6.5%) and pharmacy records (n=9, 4.2%) contributed to a smaller proportion of analyses. The largest proportion of other sources from articles with ‘other data source’ classifications included immunization records (n=12), chart reviews (n=8), and medical evacuation records (n=7) (data not shown).

## Bibliometric Summary

The articles published to the *MSMR* website hosted by health.mil garnered a total of 274,518 unique page views from 2019 to 2023, with a median of 296 unique page views per article. Four articles received over 10,000 web page views, with Testosterone Replacement Therapy Use Among Active Component Service Men exceeding all other articles during the 5-year period for maximum unique views (n=41,167) (**Table 2**). The publication date of that article (March 1, 2019) corresponds with a 2018 report from the U.S. Department of Veterans Affairs Office of the Inspector General that documented health care providers’ poor adherence to guideline recommendations for the diagnosis and treatment of men with hypogonadism. [3]

The National Center for Biotechnology Information LinkOut service routinely tracks the number of clicks from the *MSMR* publisher icon in PubMed’s abstract display to the journal’s open access, full text articles on health.mil. [4] The 2023 *MSMR* LinkOut clicks from PubMed remained relatively stable compared to 2022 (**Figure 1**). Over the 5-year publication period, however, the number of LinkOut clicks almost doubled, from 2,271 to 4,160. The far greater number of total page views of articles hosted on the *MSMR* health.mil website compared to LinkOut clicks from PubMed indicates a significant readership originating from the AFHSD journal home page.

*MSMR* also tracks CiteScore metrics from Scopus, which are based on the number of citations to articles published by a journal over 4 years, divided by the number of the same document types indexed in Scopus and published during those respective 4 years. [5] The *MSMR* CiteScore has continually increased over the past 3 years, from 0.7 in 2020 to 1.9 in 2022.

## Future Direction

While *MSMR* will continue to maintain full text, open access to articles through publication on the journal website in 2024, the full text access from the PubMed abstract display will begin linkage to PubMed Central. This full text archival process will potentially expand readership to a larger academic community, while standardizing and improving historic archival links to PubMed. The journal will also continue to track CiteScore metrics from Scopus. The improvement of the CiteScore metric in recent years corresponds with the increasing number of PubMed LinkOut clicks over the same period, which may be further bolstered by full text archival processes to PMC during 2024.

Biosurveillance remains a high-priority mission for the DOD, with DHA Public Health prioritizing capabilities to support a better biodefense posture in 2023 and beyond. [6,7] The *MSMR* welcomes new submissions accordant with the 2023 DOD Biodefense Posture Review, which outlines significant reforms for a resilient force to deter use of bioweapons, rapidly respond to natural outbreaks, and minimize global risk of laboratory accidents. [8]

Just one-fifth of the analytic reports (i.e., full reports, brief reports, Surveillance Snapshots) published in *MSMR* from 2019 to 2023 were supported by laboratory capabilities. Critical topics such as antimicrobial resistance, wastewater surveillance, and other environmental threats were presented but are likely under-represented, as sustaining and strengthening U.S. deterrence of the biothreat environment, including naturally occurring, accidental, and deliberate biological threats, is a recently heightened priority. [7]

A substantial number of articles published from 2019 to 2023 employed dual or multi-sourced data approaches, typically combining laboratory, RME, or administrative records; pharmacy records, however, contributed to relatively few full and brief reports and Surveillance Snapshots. Pharmacosurveillance offers a different and useful perspective for public health capabilities, supporting surveillance for empirical treatment in the absence of laboratory confirmation often corresponding with lags in illness reporting. While laboratory records available within the MHS are usually limited to results generated from military hospitals and clinics, the Pharmacy Data Transaction Service (PDTS) offers a comprehensive data source for DOD beneficiaries with prescription orders originating from military hospitals and clinics, mail order, and retail-dispensed facilities. [9]

The low number of outbreak reports and case reports published over the last 5 years also indicates an opportunity to broaden content from clinicians and each of the Public Health Defense Centers engaging in local force health protection. Additionally, increased publication of editorials, letters to the editor, and notices to readers may offer another venue for publishing efforts by the Army, Navy, and Air Force to enable a healthy, ready Force.

 As we usher in a new year that will see the start of *MSMR*’s 30th year of production, our editorial staff continues to welcome new submissions, especially those aligned with DHA Public Health’s strategic position for meeting the needs of the MHS, the military services, and the Combatant Commands, for the support of our nation’s security. Detailed instructions for prospective authors are available on the *MSMR* website.

## Figures and Tables

**Table 1:** MSMR Article Content Themes by ICD Chapter Topic, 2019–2023

Topic	No.	%	** ICD Chapter Topic**		
Certain infectious and parasitic diseases	73	29.4
Injury, poisoning, and certain other consequences of external causes	19	7.7
Mental, behavioral and neurodevelopmental disorders	18	7.3
Diseases of the musculoskeletal system and connective tissue	14	5.6
Diseases of the eye and adnexa	10	4.0
Endocrine, nutritional and metabolic diseases	8	3.2
Diseases of the respiratory system	5	2.0
Diseases of the digestive system	5	2.0
Diseases of the skin and subcutaneous tissue	5	2.0
Symptoms, signs and abnormal clinical and laboratory findings, not elsewhere classified	5	2.0
Neoplasms	3	1.2
Diseases of the blood and blood-forming organs and certain disorders involving the immune mechanism	2	0.8
Diseases of the nervous system	2	0.8
Diseases of the circulatory system	1	0.4
Diseases of the genitourinary system	1	0.4
Diseases of the ear and mastoid process	0	0.0
Pregnancy, childbirth and the puerperium	0	0.0
Certain conditions originating in the perinatal period	0	0.0
Congenital malformations, deformations and chromosomal abnormalities	0	0.0
** Contact with health services or procedures (Z-codes)**		
Immunization	14	5.6
Other Z-codes	10	4.0
** Topics not aligned with ICD chapters**		
Overall health care burden or utilization	42	16.9
Public health surveillance methods	6	2.4
Other	5	2.0
**Total**	248	100.0

Abbreviation: ICD, International Classification of Diseases.

**Table 2:** 10 Most-Read Articles on the MSMR Website,2019-2023^a^

**MSMR Volume**	**Article Title**	**Unique Page Views**
MSMR Vol. 26 No. 3 (Posted Mar 1, 2019)	Testosterone Replacement Therapy Use Among Active Component Service Men, 2017	41,167
MSMR Vol. 28 No. 1 (Posted Jan 1, 2021)	The Prevalence of Attention-Deficit/Hyperactivity Disorder (ADHD) and ADHD Medication Treatment in Active Component Service Members, U.S. Armed Forces, 2014–2018	28,313
MSMR Vol. 26 No. 3 (Posted Mar 1, 2019)	Sexually Transmitted Infections, Active Component, U.S. Armed Forces, 2010–2018	13,084
MSMR Vol. 26 No. 8 (Posted Aug 1, 2019)	Update: Routine Screening for Antibodies to Human Immunodeficiency Virus, Civilian Applicants for U.S. Military Service and U.S. Armed Forces, Active and Reserve Components, January 2014–June 2019	12,022
MSMR Vol. 26 No. 3 (Posted Mar 1, 2019)	Vasectomy and Vasectomy Reversals, Active Component, U.S. Armed Forces, 2000–2017	8,078
MSMR Vol. 26 No. 3 (Posted Mar 1, 2019)	Brief Report: Male Infertility, Active Component, U.S. Armed Forces, 2013–2017	6,058
MSMR Vol. 26 No. 7 (Posted Jul 1, 2019)	Infectious Mononucleosis, Active Component, U.S. Armed Forces, 2002–2018	5,583
MSMR Vol. 26 No. 4 (Posted Apr 1, 2019)	Update: Heat Illness, Active Component, U.S. Armed Forces, 2018	5,558
MSMR Vol. 27 No. 2 (Posted Feb 1, 2020)	Images in Health Surveillance: Skin Rashes in Children Due to Infectious Causes	5,541
MSMR Vol. 26 No. 12 (Posted Dec 1, 2019)	Prevalence of Glucose-6-Phosphate Dehydrogenase Deficiency, U.S. Armed Forces, May 2004–September 2018	5,172

^a^Webpage views represent cumulative, unique views of articles published to the MSMR web site, summarized as of December 13, 2023; thus, the number of page views is expected to rise over time.

## AUTHOR AFFILIATIONS

Epidemiology and Analysis Branch, Armed Forces Health Surveillance Division, Defense Health Agency, Silver Spring, MD: Ms. Rossi, Mr. Pursley
